# Computer Vision System for Expressing Texture Using Sound-Symbolic Words

**DOI:** 10.3389/fpsyg.2021.654779

**Published:** 2021-10-07

**Authors:** Koichi Yamagata, Jinhwan Kwon, Takuya Kawashima, Wataru Shimoda, Maki Sakamoto

**Affiliations:** ^1^Graduate School of Informatics and Engineering, The University of Electro Communications, Chofu, Japan; ^2^Department of Education, Kyoto University of Education, Kyoto, Japan

**Keywords:** texture, sound-symbolic words, tactile sensation, onomatopoeia, image databases

## Abstract

The major goals of texture research in computer vision are to understand, model, and process texture and ultimately simulate human visual information processing using computer technologies. The field of computer vision has witnessed remarkable advancements in material recognition using deep convolutional neural networks (DCNNs), which have enabled various computer vision applications, such as self-driving cars, facial and gesture recognition, and automatic number plate recognition. However, for computer vision to “express” texture like human beings is still difficult because texture description has no correct or incorrect answer and is ambiguous. In this paper, we develop a computer vision method using DCNN that expresses texture of materials. To achieve this goal, we focus on Japanese “sound-symbolic” words, which can describe differences in texture sensation at a fine resolution and are known to have strong and systematic sensory-sound associations. Because the phonemes of Japanese sound-symbolic words characterize categories of texture sensations, we develop a computer vision method to generate the phonemes and structure comprising sound-symbolic words that probabilistically correspond to the input images. It was confirmed that the sound-symbolic words output by our system had about 80% accuracy rate in our evaluation.

## Introduction

Recent years have witnessed remarkable advances in machine learning. One important breakthrough technique is known as “deep learning,” which uses machine learning algorithms that automatically extract high-level features in data by employing deep architectures composed of multiple non-linear transformations. Unlike conventional machine learning methods, deep learning is similar to the human brain, which is organized as a deep neural network and processes information through multiple stages of transformation and representation. By exploiting a deep neural network to learn features at multiple levels of abstraction from data automatically, deep learning methods enable a system to perform highly complex functions that directly map raw sensory input data to the output without human manipulation. Many recent studies have reported excellent performance by applying deep learning techniques to a variety of applications, including speech recognition (Dahl et al., 2012; Hinton et al., 2012; Graves et al., 2013; LeCun et al., 2015) and natural language processing (Collobert and Weston, 2008; Conneau et al., 2016; Goldberg, 2016; Manning, 2016). Among others, remarkable advancements have been achieved in the field of computer vision using deep convolutional neural network (DCNNs). Convolutional neural networks (CNNs) combined with large-scale datasets such as ImageNet (Russakovsky et al., 2014) have made great progress in object and material recognition as well as scene classification. Unlike conventional machine learning, the effective features of an image can be automatically and quantitatively extracted in the learning process when using CNNs (Krizhevsky et al., 2012; Girshick et al., 2014; Sermanet et al., 2014; Simonyan and Zisserman, 2014; Zeiler and Fergus, 2014; Szegedy et al., 2015). Therefore, CNNs have received a large amount of attention in the general object recognition field since the ImageNet Large Scale Visual Recognition Challenge in 2012, and many CNN architectures (e.g., VGG, GoogLeNet, R-CNNs, and Over-feat) have demonstrated excellent performance in object recognition and scene classification (Girshick et al., 2014; Sermanet et al., 2014; Simonyan and Zisserman, 2014; Szegedy et al., 2015). In material recognition, DCNN features also achieved excellent performance. Cimpoi et al. (2014) proposed representing material images with state-of-the-art image representations, improved Fisher vectors (Perronnin et al., 2010), and DCNN features extracted by DeCAF (Donahue et al., 2014), and achieved a recognition rate of 67.1% for 10-class material photo classification of the Flickr Material Database (FMD). In recent years, Google obtained state-of-the-art results (an error rate of 6.6%) in the field of object category recognition in the 2014 ImageNet Large Scale Visual Recognition Challenge. In addition, Microsoft Research Asia (MSRA) achieved an error rate of 3.5% in the same contest. Furthermore, effective methods for learning such as dropout have been reported (Srivastava et al., 2014). Cimpoi et al. (2016) employed very deep CNNs for material recognition and achieved a recognition rate of 82.2% on FMD and 75.5% on the Describable Texture Dataset (DTD).

Various computer vision applications using DCNNs have been employed in domains such as self-driving cars, facial and gesture recognition, and automatic number plate recognition. Despite the recent advances in material recognition, high-level human cognition such as texture remains one of the most challenging open problems. It is still difficult for computer vision methods to express texture like a human because texture you feel from materials has no correct or incorrect answer. For example, the answer to the question “what’s this” for a cat is supposed to be “a cat.” On the other hand, the answer to the question “how do you feel the texture of a cat” could be light and feathery or warm and fluffy. Inspired by the successes of deep learning, in this paper, we attempt to develop computer vision that expresses the texture of materials in the sense of “shitsukan” introduced by Komatsu and Goda (2018). Texture in the sense of “shitsukan” is not confined to material property and surface quality as well as the feel of a finish or texture. It is also related to how you feel about the object. In the words of Komatsu and Goda (2018), Shitsukan (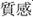) is a Japanese word whose literal meaning is the sense (kan, 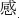) of quality (shitsu, 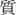), and it is commonly used to cover the wide range of topics to which material perception in a broad sense is assigned. Although every sensory modality is involved in material perception, we will focus mainly on it through vision.

Shitsukan perception is achieved by a process in which the various physical quantities (e.g., surface shape and color) of objects are detected by human sensory receptors and perceived in the brain. Material and texture perception have been studied in various fields such as neuroscience, psychophysics, and vision psychology and has been revealed to involve glossiness, transparency, wetness, and roughness perceptions (Tamura et al., 1978; Lederman et al., 1986; Whitaker et al., 2008; Bensmaia, 2009; Lederman and Klatzky, 2009; Tiest, 2010). Although a human perceives texture almost unconsciously and expresses it easily, no computer system can express the texture of materials as richly as humans. In this paper we developed a computer vision system that expresses texture using sound-symbolic words (SSWs). SSWs, or onomatopoeia, in Japanese, can describe differences in texture sensation at a fine resolution. For example, Japanese has more than 300 touch-related SSWs, more than twice the number of adjectives that describe touch experiences (Sakamoto and Watanabe, 2013). The texture of materials is not represented by a single texture-related adjective. That is, a product or material is usually expressed by two or more texture-related adjectives, while it can be expressed by only one SSW. For example, the texture of a down quilt can be expressed as softness and a light and fluffy texture, while it can be expressed by one SSW such as “fuwa-fuwa.” The texture of sand paper will be expressed as a dry and rough texture, while it can be expressed by one SSW “zara-zara.” “Sara-sara” and “zara-zara,” which are different only in the first syllable of the repetition unit, denote totally different tactile sensations. While the former is used for expressing dry but smooth and pleasant touch, the latter is used for expresses dry, rough and unpleasant touch.

In recent years, research interest has been growing in the relationship between sound symbolism and perceptual matching (Parise and Spence, 2012; Bremner et al., 2013; Sucevic et al., 2013; Revill et al., 2014; Supeviü et al., 2015). Many researchers have studied sound symbolism as an integrated expression of texture and have verified its effectiveness (Sakamoto and Watanabe, 2016, 2017, 2018; Sakamoto et al., 2016; Doizaki et al., 2017). Sakamoto and Watanabe (2018), for example, shows that for vowels, positive tactile ratings were associated with the back vowel (/u/), while negative ratings were associated with the front vowels (/i/and/e/). The central vowels (/o/and/a/) were mainly associated with rough, hard, and dry feelings. Consonants were categorized based on vocal features and articulation. The category of the voiced consonants (e.g., /dz/and/g/) corresponded to feelings of roughness, while that of voiceless consonants (e.g., /ts/,and/s/) corresponded to feelings of smoothness. The categories of the bilabial plosive (/p/and/b/) and voiced alveolar nasal (/n/) consonants were mainly related to soft, sticky and wet feelings, while that of voiceless alveolar affricate (//ts/) and voiceless velar plosive (/k/) consonants were related to hard, slippery and dry feelings. Kitada et al. (2021) conducted a functional magnetic resonance imaging experiment and showed that the brain regions engaged in tactile texture processing of object properties are also involved in mapping sound symbolic information with tactually-perceived object properties. The existence of SSWs has been demonstrated in a wide variety of languages (Köhler, 1929; Sapir, 1929; Bolinger, 1950; Hinton et al., 1994; Nuckolls, 1999; Ramachandran and Hubbard, 2001; Schmidtke et al., 2014). For example, English words starting with “sl-” such as “slime,” “slush,” “slop,” “slobber,” “slip,” and “slide” symbolize something smooth or wet (Bloomfield, 1933). Although various assumptions have been proposed for how linguistic phoneme are associated with certain stimuli and there is still no generally consensus (Sidhu and Pexman, 2018), we assume that learning process may be involved in the associative relationship between speech sounds and sensations. For example, Japanese repeatedly hear the phoneme “s” in “sara-sara” to express dry but pleasant textures, and the phoneme “s” in “sube-sube” to express dry but smoother ones. Interestingly, the phoneme with “s” is associated with smooth in English as exemplified “slippery,” “slime,” “slush,” “slop,” and “slide.” As another example, the phoneme “gl” is associated with something bright in Japanese such as “gila-gile.” In the same way, roughly half of the English words starting with “gl-” imply something visual and bright, as in glance, glare, gleam, glimmer, glamor, glass, glaze, glimpse, glint, glisten, glitter, globe, glossy, and glow (Crystal, 1995). Based on this repetition, we may learn what kind of sensation tends to be associate with certain phonemes. In other words, we assume that the human brain has a database of phonemes and perceptual learning. In this study, therefore, we aim to extract textural expressions using the learning process of sound symbolism and perceptual characteristics. More specifically, we develop a computer vision system using a DCNN that expresses the texture of materials using texture terms. To achieve this goal, we use Japanese SSW, which can describe differences in texture sensation at a fine resolution and are known to have a strong and systematic association between phonemes and texture sensations. In this study, we aim to generate textural expressions using DCNN and SSWs as variables to converge the various material and texture features.

The specific contributions of this paper are twofold. First, we created a new material image data set called the Texture-based FMD and collected SSWs corresponding to the Texture-based FMD. Our material image data set is suitable for capturing texture representation and machine learning. Second, we developed a DCNN-based computer vision system that expresses the texture of materials using SSWs. This system can stochastically represent the probabilistic phonemic elements and structure, including correlation information, that comprise SSWs using a DCNN and can describe various textures of materials and objects. The integrated expression of the texture through SSWs is a challenge for texture and material expression in the future.

In the remainder of this paper, we reported related works about material datasets in section “Material Datasets,” and we describe the new material image data set and learning model of the DCNN in section “Materials and Methods”, then we describe the results in section “Results.” We then validate our model by the accuracy rate of SSWs output by the system for images in section “Accuracy Evaluation”.

## Material Datasets

For material and texture perception, there have been two major approaches to creating material datasets. First, researchers published datasets that were focused on a single material representation created under controlled conditions (Dana et al., 1999; Hayman et al., 2004; Caputo et al., 2005; Liu et al., 2013). These datasets’ samples are photographed under various lighting conditions, viewing angles, and scales. However, these datasets are not enough to generalize material representation under complex real-world conditions because the material instances are only measured under controlled illumination or lab environments. Dana et al. (1999) created the CUReT database, which contains 61 different texture and material samples photographed in 205 different lighting and viewing conditions. The CUReT dataset was the first large-scale texture and material dataset and has become the standard for evaluating three-dimensional texture classification algorithms. This database was used for instance-level texture or material classification tasks (Leung and Malik, 2001; Varma and Zisserman, 2005). Hayman et al. (2004) extended the CUReT database to the KTH-TIPS database by adding scale variation and imaging 10 categories from the CUReT dataset at different scales. They varied the distance of the acquired sample to the camera to consider the scale of the textures and to change viewpoint and illumination angles. Subsequently, KTH-TIPS2 was introduced by Caputo et al. (2005) and contains 4,608 images from 11 material and texture categories, where each category has four samples. KTH-TIPS2 increased the intra-class variation by photographing images under a variety of conditions. Specifically, all the samples are imaged under various lighting conditions (from the front, side at 45°, and top at 45° and under ambient light), viewing angles (frontal, rotated 22.5° left, and 22.5° right) and scales (nine scales equally spaced logarithmically over two octaves). The KTH-TIPS2 database has been used for studying material recognition because it represents novel instances of materials. The limitations of all these databases are the limited measurements and acquisition under controlled lab environments. Therefore, the variation and complexity of material and texture of real-world scenes are not included within them. In contrast, the second category of datasets is characterized by acquisition under uncontrolled conditions (Sharan et al., 2009; Cimpoi et al., 2014). For instance, researchers have created datasets using images from an Internet image database such as Flickr. The datasets have the merits that the intra-class variance of materials and the environmental conditions can be considered.

The FMD was created to represent the large intra-class variation of materials in complex real-world scenes (Sharan et al., 2009). The FMD consists of Flickr photos downloaded from Flickr.com and material samples under uncontrolled illumination and viewing conditions. It contains 1,000 images from ten common material categories: “Fabric,” “Foliage,” “Glass,” “Leather,” “Metal,” “Paper,” “Plastic,” “Stone,” “Water,” and “Wood.” The 100 color photographs in each category are characterized by 50 close-ups and 50 object-level views. These images capture the diversity of real-world material appearance by avoiding the poor intra-class variation found in earlier databases. In addition, Cimpoi et al. (2014) released the DTD, which includes 5,640 texture images representing real-world texture images. They annotated the texture images with one or more adjectives (describable texture attributes) selected in a vocabulary of 47 English words such as banded, chequered, dotted, fibrous, grid, meshed, and zigzagged. They used a crowd-sourcing service, Amazon Mechanical Turk, to select good images from images gathered from the Web. DTD addresses how the problem of texture description differs from those of material recognition considered in CUReT, KTH, and FMD. Specifically, they addressed the fact that the describable attributes depend on subjective properties such as human judgments, whereas materials are objective. However, the objects we encounter in every-day life are hardly represented by a single adjective (describable texture attributes) because they usually have two or more attributes. In addition, the GeoMat dataset, which provides real world material images and geometric information and the Materials in Context database, which consists of many full scenes with material labels, are also available (Bell et al., 2015; DeGol et al., 2016).

## Materials and Methods

### Texture-Based Flickr Material Database

In this study, we focus on FMD, which contains the diversity of real-world material appearances and has been acquired under uncontrolled conditions (Sharan et al., 2009). However, it is difficult to describe and extract the texture features from FMD images because multiple objects and textures are included in the images. In fact, there are four spatial scales for visual recognition: surface (extreme close-up views), material (close-up views), object (regular views), and scene (zoomed-out views), but the FMD depicts spatial scales in the range from material (close-up views) to scene (zoomed-out views) (Sharan et al., 2013). Therefore, in this study, we created a new image dataset suitable for texture and deep learning. To create this new dataset, we conducted an experiment to identify texture images from FMD images.

**FIGURE 1:**
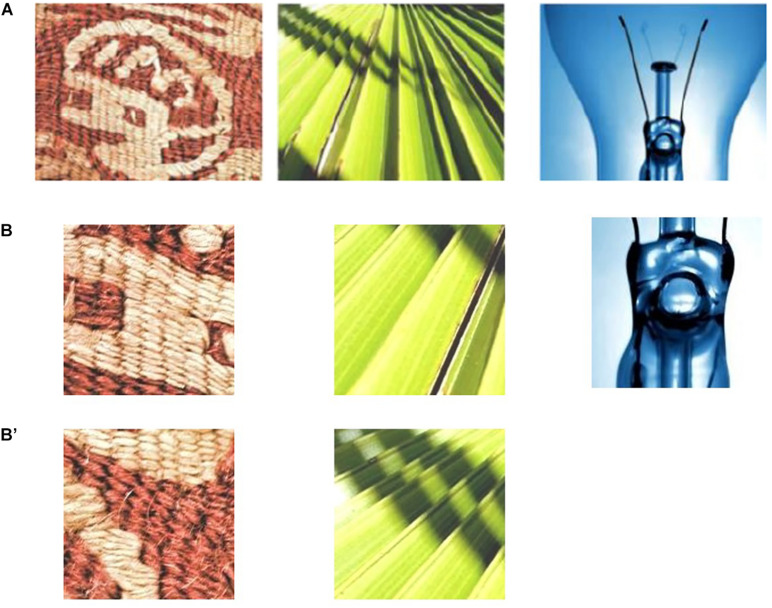
**(A)** Examples of FMD images and **(B,B′)** examples of extracted FMD texture images.

One hundred participants (25 women and 75 men, mean age 22.1 years) participated in the experiment and were divided into 10 groups. All participants had normal hearing and normal or corrected-to-normal visual acuity, and were not informed of the purpose of the experiment. Participants were paid to take part in the experiments, and written informed consent was obtained. These experiments were approved by the ethics committee of the University of Electro-communications. We divided all 100 images in each 10 material categories (fabric, foliage, glass, leather, metal, paper, plastic, stone, water, and wood) into 10 groups. As a result, 1,000 FMD images were classified into 10 groups. Figure 1A shows an example of the FMD image stimuli. Each group of visual stimuli were presented for each participant group. Each trial was conducted in an isolated test room under controlled lighting conditions. Participants were kept at a viewing distance of approximately 50 cm from a touch panel display showing the visual stimuli. The visual stimuli were presented vertically at eye height in a random order using the slideshow function of Microsoft Power-Point 2010. Participants were given a brief explanation of SSWs with some examples of SSWs that were assumed to be used for texture. Participants were asked to describe texture spontaneously using SSWs and mark the part of the visual stimulus they focused on when describing the texture. We then cropped each image part that three or more participants marked on each image to produce a new image set. Figures 1B,B′ show examples of cropped images. For example, two images were cropped from the left and center images of Figure 1A, respectively, and one image was cropped from the right image of Figure 1A. Because the average size of the image parts marked by participants was approximately 100 pixels, we cropped square images of 150 × 150 pixels from the original images of 512 × 384 pixels. We obtained about 2 cropped images from each original image. Consequently, we obtained a total of 1,946 image samples.

### System That Expresses Texture Using Sound-Symbolic Words Corresponding to the Texture-Based Flickr Material Database

In this section, we report the results of an experiment to investigate SSWs corresponding to the Texture-based FMD. Because texture representations can be expressed by a variety of SSWs by various phonemes, and because there is not one correct answer, we asked 10 subjects per image to describe texture spontaneously using SSWs. One hundred participants (25 women and 75 men, mean age 20.6 years) took part in the experiment and were divided into 10 groups. All participants had normal hearing and normal or corrected-to-normal visual acuity, and were not informed of the purpose of the experiment. Participants were paid to take part in the experiments, and written informed consent was obtained. These experiments were approved by the ethics committee of the University of Electro-communications. The apparatus and procedure were the same as in the above experiment with the following exceptions: We used the newly created 1,946 image samples, which were classified into 10 groups. Each group of visual stimuli was presented to each participant group. Participants were given a brief explanation of SSWs with some examples of SSWs that were assumed to be used for texture. Participants were instructed to spontaneously describe the texture of the material shown in each image using one to six SSWs. As a result, we obtained 29,443 SSW tokens (1,885 different SSWs). Table 1 shows SSWs described freely by the participants for 20 images out of 1,946 images used in this experiment. The numbers in this table mean the numbers of times the SSWs were described for each image. Some of the them are commonly used, such as “gowa-gowa” and “sara-sara,” while some are unfamiliar and unique, such as “moyo-moyo” and “kiri-kiri”.

**TABLE 1:** SSWs described freely by the participants in the experiment conducted in section “System That Expresses Texture Using Sound-Symbolic Words Corresponding to the Texture-Based Flickr Material Database”.

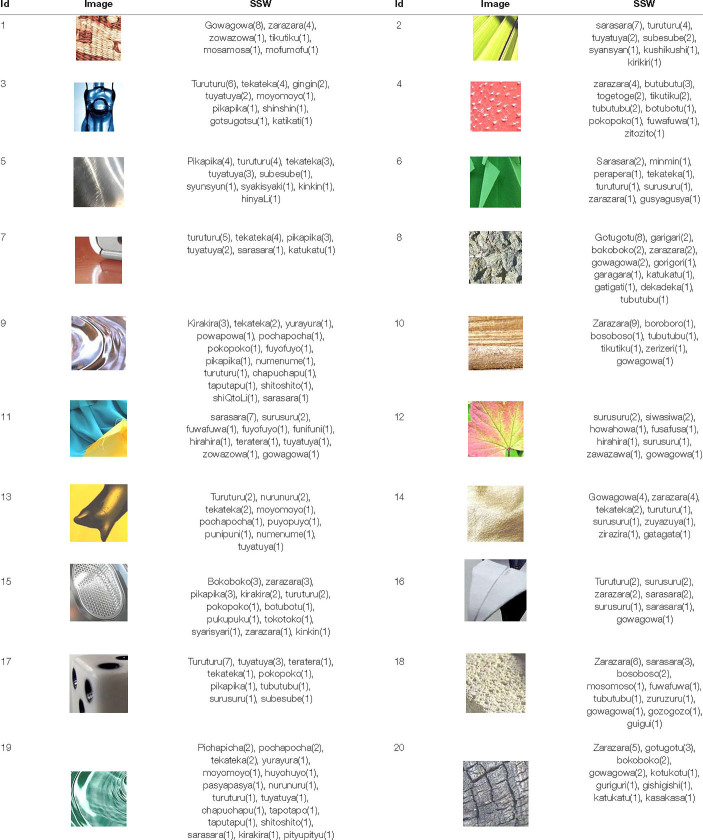

*The numbers mean the number of times the SSWs were described.*

### Learning Model

When detailed textures are freely expressed by SSWs, there is no single correct answer, and a variety of SSWs can be used. The probability distributions of phonemes in SSWs used here can be expected to contain detailed information about the texture. In this paper, we construct a learning model that estimates the probability distribution of phonemes in SSWs for images to construct a system to generate SSWs.

The training data are pairs of images and phonetic binary vectors of SSWs, and we developed a model that predicts phonological probability vectors from images. The phonological vectors consist of 5, 27, 3, 5, 27, 4, 8, 1, and 16 dimensional binary vectors v1, c1, s1, v2, c2, s2, r, and h (see Table 2). The five-dimensional hot-one vectors v1 and v2 correspond to the vowels of the first and second phonemes, respectively. The 27-dimensional hot-one vectors c1 and c2 correspond to the consonants of the first and second phonemes, respectively. The binary vectors s1 and s2 represent the presence or absence of special phonemes following the first and second phonemes. The single binary value r represents the presence or absence of repetition. Since our model estimates each probability of phonological element independently, above vectors alone do not reflect the probabilistic correlations. To account for probabilistic correlations, we added a 16-dimensional binary sequence h generated by hashing v1, c1, s1, v2, c2, s2, and r using MD5. Since MD5 breaks independence uniformly, we can expect h to contain correlation information.

**TABLE 2:** 88-Dimensional SSW array.

Phonological characteristics	Dimensions	Phonemes
Vowels 1 (v1)	5	/a/,/i/,/u/,/e/,/o/
Consonants 1 (c1)	27	/k/,/ky/,/g/,/gy/,/s/,/sy/,/z/,/zy/,/t/,/ty/, /d/,/dy/,/n/,/ny/,/h/,/hy/,/b/,/by/,/p/,/py/, /m/,/my/,/y/,/r/,/ry/,/w/or absence
Special phonemes 1 (s1)	3	/N/,/Q/,/R/
Vowels 2 (v2)	5	/a/,/i/,/u/,/e/,/o/
Consonants 2 (c2)	27	/k/,/ky/,/g/,/gy/,/s/,/sy/,/z/,/zy/,/t/, /ty/,/d/,/dy/,/n/,/ny/,/h/,/hy/,/b/,/by/, /p/,/py/,/m/,/my/,/y/,/r/,/ry/,/w/or absence
Special phonemes 2 (s2)	4	/N/,/Q/,/R/,/Li/
Repetition (r)	1	Presence or absence
MD5 (h)	16	Binary sequence

**FIGURE 2:**
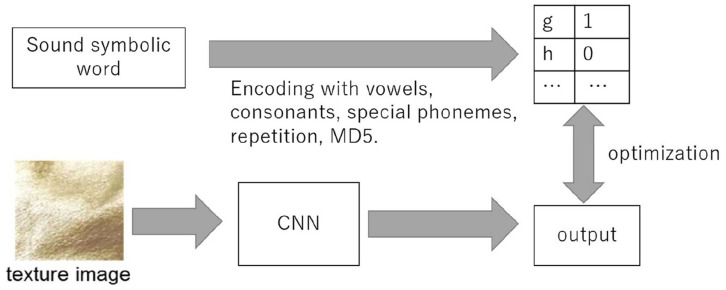
Overview of our method.

Figure 2 shows the overview of the learning model used in our study. This model takes images as input and outputs 88-dimensional vectors to estimate phonological vectors. We used VGG 16, a learning model of 1,000 types of general object recognition performed in 2015, as a reference CNN model, which contains 13 CONV layers and 3 FC layers (Simonyan and Zisserman, 2014). In addition, we applied dropout to the first two FC layers to avoid overfitting with a dropout ratio set to 0.5. The output vectors are divided into 5, 27, 3, 5, 27, 4, 8, 1, and 16 dimensional vectors, and the loss function is the sum of loss functions for phonological vectors v1, c1, s1, v2, c2, s2, r, and h. The log-softmax loss function is used for v1, c1, v2, c2, and the log-sigmoid loss function is used for s1, s2, r, h. Note that the total cross entropy loss for several independent models is the sum of the cross-entropy losses for each model. We used stochastic gradient descent and mini-batch training with a batch size of 40 and a momentum factor 0.5. We set the learning coefficient parameter to 0.0000025, number of epochs to 1,00, and CONV layers were frozen. The 1,946 images were divided into 1,751 training data and 195 test data for 10-fold cross-validation.

## Results

**FIGURE 3:**
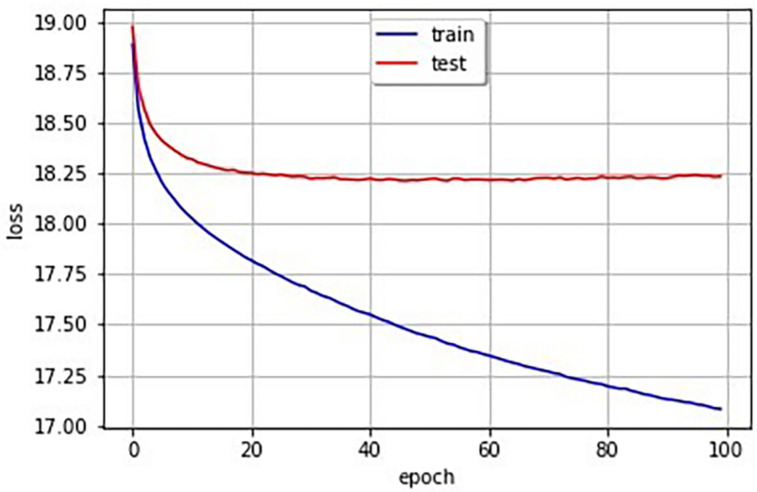
Learning curve (the blue line is training data and the red line is test data).

Figure 3 shows the learning curves obtained by the learning model with 29,443 pairs of images and phonetic binary vectors of SSWs. The decrease of cross-entropy losses can be confirmed.

The aim of this research is to output the stochastic phonemes describing the material textures. Table 3 shows the estimated probability vectors for the phonemes with the exception of MD5, which were output when the leftmost image in Figure 1B was input to the model. This system can stochastically represent the output frequency of the phonemes and structure comprising the SSW. In particular, if we select and combine phonemic and structural elements with the highest number of occurrences, the SSW becomes “gowa-gowa.” In fact, “gowa-gowa” was the most frequently answered SSW by the participants. Furthermore, when we used the next highest numbers of occurrences, we obtained the SSWs “zara-zara,” “mosa-mosa,” and “boko-boko.” This probabilistic phoneme output led to a new method that expressed the diversity of a material’s texture. The output corresponding to MD5 can exclude undesirable combinations of phonemes such as “zowo-zowo” and “garu-garu,” because cross-entropy losses of these SSWs for vector h are high.

**TABLE 3:** Example of output array translated to normalized weight by softmax and sigmoid functions (S/P is the special phoneme category and the numerical values show the output frequency rates).

First mora						Second mora						Repetition	
Vowel		Consonant		S/P		Vowel		Consonant		S/P			
O	0.56	g	0.34	N	0.02	a	0.75	w	0.38	N	0.01	Re	0.96
A	0.29	z	0.22	Q	0.01	o	0.11	r	0.19	Q	0.02		
U	0.08	m	0.15	R	0.01	u	0.07	s	0.13	R	0.01		
I	0.05	b	0.10			i	0.05	k	0.09	L	0.01		
…	…	…	…			…	…	…	…				

## Accuracy Evaluation

**FIGURE 4:**
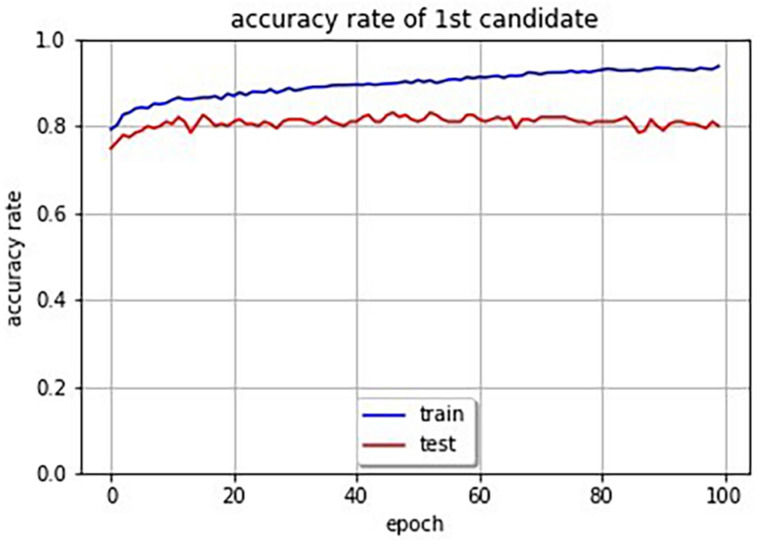
Accuracy rate curves of first candidate SSWs (the blue line is training data and the red line is test data).

**FIGURE 5:**
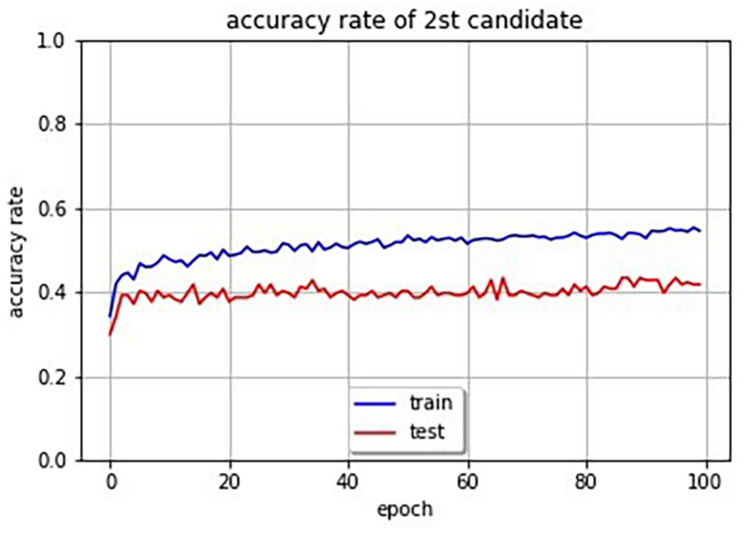
Accuracy rate curves of second candidate SSWs (the blue line is training data and the red line is test data).

In order to evaluate the accuracy of the system, we attempted to obtain the accuracy rate of SSWs output by the system for images. Since there are a huge number of possibilities of combination of phonemes for SSWs, and since there is no one right answer for an image, some restrictions and rules are necessary. We restricted SSWs to the 1,885 different SSWs that were answered in the experiment. Our system can compute cross entropy loss for an image and SSW pair. Therefore, we selected the SSW with the lowest loss in the 1,885 SSWs for a given image as the first and second candidate SSWs. Furthermore, we defined the candidate SSW is correct if it is included in the SSWs answered in the experiment for the given image. Table 4 shows SSWs generated by the system for some images that is not used for the learning. These are the three candidate SSWs with the smallest cross entropy loss for each image. Each generated SSW is marked by asterisk and considered the correct answer if the same SSW was answered in the experiment conducted in section “System That Expresses Texture Using Sound-Symbolic Words Corresponding to the Texture-Based Flickr Material Database.” The curves in Figure 4 shows the accuracy rate of the first candidate SSWs given by our system. We can see that even the test images, which is not used for training, have accuracy rates of about 80%. In the same way, we computed the accuracy rates of the second candidate SSW. The curves in Figure 5 shows the accuracy rate of the second candidate SSWs given by our system. We can confirm that the second candidate SSW has about 40% of accuracy rates.

**TABLE 4:** SSWs generated by the system for some images that is not used for the learning.

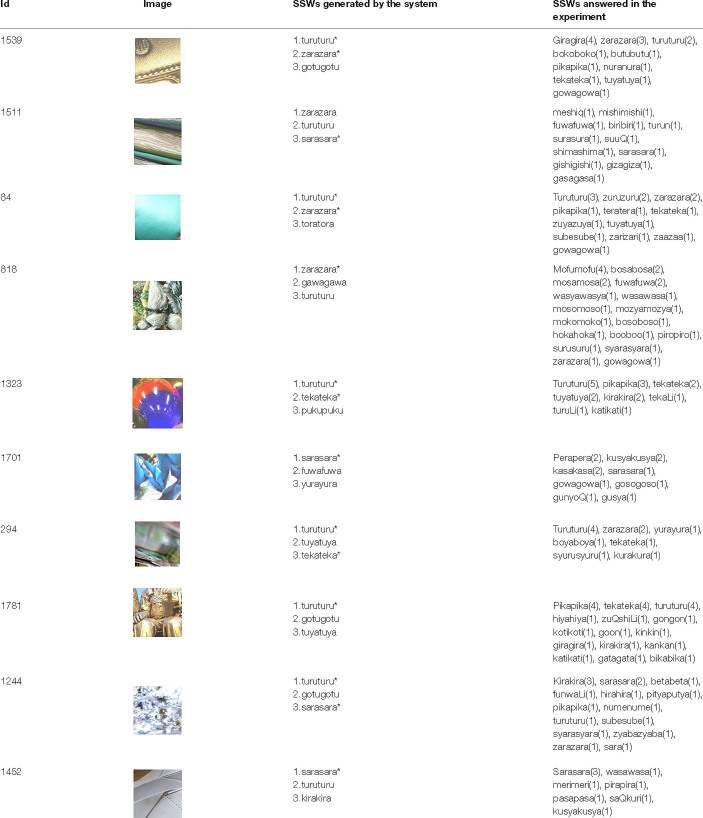

*These are the three SSWs with the smallest cross entropy loss for each image. Each generated SSW is marked by asterisk if the same SSW was answered in the experiment conducted in section “System That Expresses Texture Using Sound-Symbolic Words Corresponding to the Texture-Based Flickr Material Database”.*

## Conclusion and Future Work

In this paper, we developed a DCNN-based computer vision system that expresses the texture of materials. To achieve this goal, we used Japanese SSW expressing texture, which can describe differences in texture sensation at a fine resolution and are known to have strong and systematic associations between perceptual sensations and phoneme. As a result, it became possible to stochastically represent the output frequency of the phonemes and structure of the SSWs for the input images. It was confirmed that the SSWs output by our system had about 80% accuracy rate in our evaluation.

One application of this technique is to create new names according to the characteristics of a texture. Previous studies have pointed out that consumers associate fictitious brand names with product’s property-related information (Dana et al., 1999; Hayman et al., 2004). For example, a previous study demonstrated that people expected a creamier, richer, and smoother ice cream when it was named “Frosh” rather than “Frish” (Caputo et al., 2005). In this study, we focused on the relationship between texture and perceptual phonemes and thus, it was possible to output perceptual phonemes according to the texture characteristics. Therefore, our concept could be applied when creating new names for new textures, materials, or new objects. It is said that humans and robots will coexist in the future. If a computer vision that can express textures like humans is created, robots equipped with such computer visions may be able to teach textures for blind people.

The limitation of this study is that it was studied only in Japanese. However, we believe that this concept could be applied to other languages in the future and the output of perceptual phonemes on images could be investigated and applied globally. In fact, the existence of sound-symbolic words (SSWs) has been demonstrated in a wide variety of languages (Köhler, 1929; Sapir, 1929; Bolinger, 1950; Hinton et al., 1994; Nuckolls, 1999; Ramachandran and Hubbard, 2001; Schmidtke et al., 2014). English words starting with “sl-” such as “slime,” “slush,” “slop,” “slobber,” “slip,” and “slide” symbolize something smooth or wet (Bloomfield, 1933). Doizaki et al. (2017) conducted an experiment at a workshop held at the World Haptics 2013 conference and observed the “bouba-kiki” effect in touch using eight tactile stimuli. Around 60 people participated in the workshop, and more than half of the participants were from Europe, the United States, and other countries. Universality of sound symbolism has been suggested by the previous researches. However, we assume that learning process may be involved in the associative relationship between speech sounds and sensations. Therefore, each language or culture may need its own database to create a computer vision expressing texture.

## Data Availability Statement

The raw data supporting the conclusions of this article will be made available by the authors, without undue reservation.

## Ethics Statement

Ethical review and approval was not required for the study on human participants in accordance with the local legislation and institutional requirements. The patients/participants provided their written informed consent to participate in this study.

## Author Contributions

KY designed the method and the system. JK and TK were responsible for much of the system development. MS was an expert in Kansei systems and her contribution to the idea was significant. All authors contributed to the article and approved the submitted version.

## Conflict of Interest

The authors declare that the research was conducted in the absence of any commercial or financial relationships that could be construed as a potential conflict of interest.

## Publisher’s Note

All claims expressed in this article are solely those of the authors and do not necessarily represent those of their affiliated organizations, or those of the publisher, the editors and the reviewers. Any product that may be evaluated in this article, or claim that may be made by its manufacturer, is not guaranteed or endorsed by the publisher.
